# Healthcare professionals’ perspectives of barriers and facilitators in implementing physical activity programmes delivered to cancer survivors in a shared-care model: a qualitative study

**DOI:** 10.1007/s00520-019-05108-1

**Published:** 2019-12-02

**Authors:** Charlotte IJsbrandy, Wim H. van Harten, Winald R. Gerritsen, Rosella P.M.G. Hermens, Petronella B. Ottevanger

**Affiliations:** 1 Radboud Institute for Health Science (RIHS), Scientific Institute for Quality of Healthcare (IQ healthcare), Radboud University Medical Center Nijmegen, PO Box 9101, 6500 HB Nijmegen, The Netherlands; 2 Radboud Institute for Health Science (RIHS), Department of Medical Oncology, Radboud University Medical Center Nijmegen, PO Box 9101, 6500 HB Nijmegen, The Netherlands; 3 Radboud Institute for Health Science (RIHS), Department of Radiation Oncology, Radboud University Medical Center Nijmegen, PO Box 9101, 6500 HB Nijmegen, The Netherlands; 4 Netherlands Cancer Institute, Division of Psychosocial Research and Epidemiology, Plesmanlaan 121, 1066 CX Amsterdam, The Netherlands; 5 Department of Health Technology and Services Research, University of Twente, MB-HTSR, PO Box 217, 7500 AE Enschede, The Netherlands

**Keywords:** Exercise, Health plan implementation, Neoplasms, Qualitative research, Rehabilitation, Cancer survivors

## Abstract

**Background:**

The positive impact of physical activity programmes has been recognised, but the current uptake is low. Authorities believe delivering these programmes in a shared-care model is a future perspective. The present study aimed to identify the barriers and facilitators affecting physical activity programme implementation in a shared-care model delivered with the cooperation of all the types of healthcare professionals involved.

**Methods:**

Thirty-one individual interviews with primary healthcare professionals (PHPs) and four focus group interviews with 39 secondary healthcare professionals (SHPs) were undertaken. We used Grol and Flottorp’s theoretical models to identify barriers and facilitators in six domains: (1) physical activity programmes, (2) patients, (3) healthcare professionals, (4) social setting, (5) organisation and (6) law and governance.

**Results:**

In the domain of physical activity programmes, those physical activity programmes that were non-tailored to the patients’ needs impeded successful implementation. In the domain of healthcare professionals, the knowledge and skills pertaining to physical activity programmes and non-commitment of healthcare professionals impeded implementation. HCPs expressed their concerns about the negative influence of the patient’s social network. Most barriers occurred in the domain of organisation. The PHPs and SHPs raised concerns about ineffective collaboration and networks between hospitals. Only the PHPs raised concerns about poor communication, indeterminate roles, and lack of collaboration with SHPs. Insufficient and unclear insurance coverage of physical activity programmes was a barrier in the domain of law and governance.

**Conclusions:**

Improving the domain of organisation seems the most challenging because the collaboration, communication, networks, and interactive roles between the PHPs and SHPs are all inadequate. Survivor care plans, more use of health information technology, improved rehabilitation guidelines, and better networks might benefit implementing physical activity programmes.

**Electronic supplementary material:**

The online version of this article (10.1007/s00520-019-05108-1) contains supplementary material, which is available to authorized users.

## Background

There is a distinct deterioration of the patient’s physical activity during cancer treatment. It is also well known that patients’ physical activity levels are generally lower even after they have completed their cancer treatment [1]. However, the positive impact of physical activity programmes on these patients has recently been recognised and has been shown to improve both psychological and physiological functions [2–6]. Therefore, evidence-based guidelines recommend physical activity programmes or the use of other initiatives to improve the uptake of physical activity during and after cancer and its treatment. Nonetheless, it appears that current uptake is low [7–10].

The success of implementing physical activity programmes depends on the care model that makes provision for these programmes. Survivor care had been traditionally delivered by secondary healthcare professionals (SHPs). However, concern exists about the sustainability of this care route in the face of the continuing increase of cancer survivors [11, 12]. A shared-care model with care partly shifted to primary healthcare professionals (PHPs) may be a solution. It might optimise adherence to guidelines for recommended follow-up and rehabilitation of cancer survivors [13]. Authorities believe that a shared-care model is needed to provide qualitative and cost-effective survivor care in the future [14, 15]. Therefore, evidence-based guidelines recommend physical activity programmes delivered in a shared-care model. In the context of our study, a shared-care model is an organisational model involving both PHPs and SHPs working together as a team. Screening, referral, and delivery of physical activity programmes should be offered partly by PHPs and partly by SHPs, depending on the needs of the cancer survivor. However, concerns exist because the collaboration between primary care and secondary care is still lagging [16]. There are many barriers, and only a few PHPs and SHPs prefer a shared-care model for cancer survivors [17–22].

Those researching the factors affecting physical activity programme implementation should also take into account the effects of delivering physical activity programmes in a shared-care model. This model might be more challenging than traditional models since it involves more healthcare professionals (HCPs) in both primary and secondary care. However, since the shared-care model for cancer survivors is the model of the future for providing screening, referral, and delivery of physical activity programmes, more insight into the barriers and facilitators affecting physical activity programme implementation in this model is needed. Therefore, the present exploratory and qualitative study aimed to identify the barriers and facilitators affecting physical activity programme implementation delivering in a shared-care model.

## Methods

Individual and focus group interviews provided data about the factors affecting physical activity programme implementation in a shared-care model delivered by PHPs and SHPs. This qualitative study was carried out following the Consolidated criteria for Reporting Qualitative studies (COREQ) [23]. We invited HCPs who should be involved in the screening, referral, and delivery of physical activity programmes in the shared-care model. Five hospitals in the Netherlands (one categorical, one university, one teaching and two non-teaching) were invited to participate in this qualitative study.

### Setting

In the Dutch healthcare system, physical activity programmes are offered by (1) rehabilitation physicians in hospitals or rehabilitation clinics; apart from physical problems, patients also need to have been diagnosed with psychological or social problems to be allowed to participate in these physical activity programmes; (2) multiple HCPs (e.g. sports-medicine physicians, physiotherapists) or by non-HCPs such as sports trainers; these programmes are mainly offered outside of the hospitals. The majority (> 70%) of patients only attends the second physical activity programmes.

The Dutch healthcare system is a managed competition based healthcare system [24]. The financing includes a mandatory universal basic health insurance that provides financial coverage of a comprehensive and uniform package of health services. Dutch residents can also obtain additional health insurance. The physical activity programmes that rehabilitation physicians offer are mainly covered by the basic health insurance, yet they are only available for cancer survivors with multidimensional problems. For the other, mostly monodisciplinary physical activity programmes the financial coverage is sometimes guaranteed by additional health insurance. These physical activity programmes need to be financed mainly by the patients themselves.

### Study population

#### PHPs

As a sampling technique we used purposeful sampling. Individual interviews were conducted with PHPs involved in the treatment of patients with cancer in primary care. The PHPs were practicing in the region of the five participating hospitals (e.g. general practitioners (GPs) and physiotherapists). The PHPs were asked to participate by letter and could reply to accept. One researcher (CIJ) phoned the PHPs who agreed to participate to inform them about the study and to answer their questions. The PHPs also received written information stating the objectives and the process of the individual interview. Then, the researcher made appointments for interviews with the participants.

#### SHPs

Four of the five hospitals (one categorical, one university, one teaching, and one non-teaching) participated in this qualitative focus group study. Seven to 13 SHPs treating cancer patients in secondary care were invited from each hospital (e.g. surgeons, radiotherapists, medical oncologists, gynaecologists, urologists, rehabilitation physicians, sports-medicine physicians, physiotherapists, physician assistants, nurses and psychologists). The SHPs were invited by e-mail, to which they could reply. Meetings were arranged in all the participating hospitals to inform the SHPs about the study and to answer their questions. Then the researcher determined the time and place of the focus group interviews.

#### Data collection

The HCPs were informed about the study, then informed consent and permission to audiotape the interviews was obtained. The HCPs were asked (also before the interviews began) to fill in a questionnaire about their age, gender, years of practice, function and specialty.

We developed three interview guides with Grol [25] and Flottorp’s [26] theoretical models for identification of factors influencing implementation of care innovations. Based on the theoretical models of Grol and Flottorp the influencing factors had been coded in the following six domains: (1) characteristics of the physical activity programmes, (2) characteristics of the patients, (3) characteristics of the HCPs, (4) characteristics of the social setting, (5) characteristics of the organisation and (6) characteristics of the law and governance.

The interviews were structured as follows: we asked the HCPs to describe their experiences with physical activity programmes delivered in a shared-care model for survivors. As soon as barriers or facilitators came up, we explored them in detail, using the theoretical frameworks. The interviews gave the HCPs a chance to talk freely, as well as to express their personal feelings about the barriers to and facilitators of optimal delivered physical activity programmes for patients with or after cancer. The individual interviews took about 30 min each and were conducted by one experienced researcher (CIJ). The focus group interviews took about 90 min each and were conducted by two experienced researchers (CIJ and RH). New interviews were performed until saturation was reached.

#### Planned analytic approach and outcomes

Al interviews were audio-taped and afterwards literally typed up verbatim in manuscripts, using Microsoft Word. These manuscripts were imported and analysed in qualitative software package Atlas.ti. We used version 7.6.16 for this purpose. The content analysis process, as described by Elo et all [27] was used as methodology for the analysis. Two researchers qualitatively and independently coded the barriers and facilitators mentioned in the manuscript of the interviews. We coded the influencing factors in one of the six domains. Identified factors previously not present in the models were added. The two researchers (CIJ and LB) discussed their interpretation until consensus was reached. This was done separately for the individual PHP interviews and the SHP focus group interviews. We compared the outcomes for the PHPs and SHPs to find similarities and differences among the barriers.

## Results

### Characteristics of the PHPs

Table 1 outlines the characteristics of the PHPs. Individual interviews were conducted with 31 PHPs, of whom 7 came from the region of the categorical hospital, 3 from the region of the university hospital, 8 from the region of the teaching hospital, and 8 and 5 from the region of the two non-teaching hospitals.

**Table 1:** Characteristics of primary healthcare professionals

Age (years)	Mean 47.5, SD (10.8), range (30–63)
	***n*(%)**
Total	31 (100)
Gender Male Female	15 (48.4) 16 (51.6)
Nationality Dutch American	30 (96.8) 1 (3.2)
Function General practitioner Physiotherapist	14 (45.2) 17 (54.8)
Years in profession < 1 year 1–2 years 2–5 years 5–10 years 10–19 years > 20 years	2 (6.5) 1 (3.2) 5 (16.1) 5 (16.1) 17 (54.8) 1 (3.2)
Type of hospital Categorical University Teaching Non-teaching 1 Non-teaching 2	7 (22.6) 3 (9.7) 8 (25.8) 8 (25.8) 5 (16.1)

The mean age of the PHPs who participated in the individual interviews was 47.5 years. Of the PHPs, 51.6% were women and 48.4% were men. Most had a Dutch background. Fourteen PHPs were GPs and 17 were physiotherapists; 74.1% of the participants had more than 5 years of professional experience.

All the physiotherapists were aware of the existence of physical activity programmes and were involved in a physical activity programme. Among the GPs, 64.3% were aware of the existence of physical activity programmes and 28.6% had previously referred patients to physical activity programmes.

### Characteristics of the SHPs

Table 2 shows the characteristics of the SHPs. The four group discussions were conducted with a total of 39 SHPs. Thirteen SHPs worked in the categorical hospital, 7 in the university hospital, 7 in the teaching hospital and 12 in the non-teaching hospital. The mean age of the SHPs who participated in the focus-groups was 43.2 years. The SHPs consisted of 82.1% women and 17.9% men. Most had a Dutch background.

**Table 2:** Characteristics of secondary healthcare professionals

Age (years)	Mean 43.2, SD (10.9), range (24–68)
	***n*(%)**
Total	39 (100)
Gender Male Female	7 (17.9) 32 (82.1)
Nationality Dutch German Non-Western Missing	36 (92.3) 1 (2.6) 1 (2.6) 1 (2.6)
Function Physician or Surgeon Paramedic Nurse Other	13 (33.3) 12 (30.6) 13 (33.3) 1 (2.8)
Years in profession < 1 year 1–2 years 2–5 years 5–10 years 10–19 years > 20 years	1 (2.6) 4 (10.3) 6 (15.3) 10 (25.6) 14 (35.9) 4 (10.3)
Name hospital Categorical University Teaching Non-teaching 1	13 (33.3) 7 (17.9) 7 (17.9) 12 (30.8)

Physicians and surgeons made up 33.3% of the SHPs involved in the focus group; paramedics, 30.6%; nurses, 33.3%; and 2.8% had a different profession (spiritual counsellor). Altogether, 71.8% of the focus group participants had more than 5 years of professional experience. All the SHPs were aware of the existence of physical activity programmes; 92.3% had referred patients to physical activity programmes, and 51.2% were involved in physical activity programmes themselves.

### Factors affecting PCRP implementation

The qualitative individual and focus group analyses uncovered a wide variety of main themes for barriers and/or facilitators in the six domains. We qualitatively analysed the interviews in two categories: (1) themes of barriers and/or facilitators reported by PHPs (outlined in Table 3) and (2) themes of barriers and/or facilitators reported by SHPs (outlined in Table 4). The most important barriers and/or facilitators, and the differences between PHPs and SHPs are outlined here.

**Table 3:** Factors affecting implementation of physical activity programmes among primary healthcare professionals

Barriers	Facilitators
Characteristics of physical activity programmes	
Insufficient evidence of effect of physical activity programmes	Peers available in physical activity programme
No consensus for content of physical activity programme	Clear goals of physical activity programme
Choice and tailoring	
- Choice in HCP providing programme, frequency, duration, format of programme, exclusive physical (exercise classes) or multidimensional programme, individual or as group sessions and inside or outside of cancer treatment facility	
- Tailored on patient characteristics: patient personality, lifestyle, cultural and economic background, age, tumour type, physical and psychosocial status	
Characteristics of patients	
Unaffordable physical activity programme	
- Patient cannot finance physical activity programme	
Too little knowledge about their own health and healthcare process	
No responsibility for their own health	
Not enough time	
Characteristics of professionals	
PHPs without enough knowledge and skills about physical activity programmes	
PHPs expecting extra work	
- PHPs expecting more time pressure due to extra work	
Non-committed HCPs	
- HCPs do not expect benefit for the patient	
- HCPs do not attend educational training about physical activity programmes	
Characteristics of the social setting and context in which the physical activity programme has to be applied	
Negative influence of social network on patient	
Characteristics of the organisation	
Not enough cooperation between healthcare institutes and HCPs	
Not enough communication with SHPs	
- GP not involved in healthcare process	
Difficulties reaching physical activity programme contact persons	
Insufficient quality assurance	
Not enough networking	
Not enough coordination	
Inadequate triage system	
Physical activity programme offered at wrong time in treatment schedule	
Physical activity programme scheduled at the wrong place and time	
Not enough information about physical activity programmes available to patient	
Limited capacity for delivering physical activity programmes	
- Limited capacity of financial sources, facilities and materials	
- Limited capacity of HCPs	
- Other projects require full capacity of available HCPs	
Characteristics of the laws and governance	
Unclear insurance	

*HCP* healthcare professional, *PHP* primary healthcare professional, *SHP* secondary healthcare professional

**Table 4:** Factors affecting the implementation of physical activity programmes among secondary healthcare professionals

Barriers	Facilitators
Characteristics of physical activity programmes	
Insufficient evidence of effects of physical activity programmes	Peers available in physical activity programme
Choice and tailoring	
- Choice in HCP providing programme, frequency, duration, format of programme, exclusive physical (exercise classes) or multidimensional programme, individual or as group sessions and inside or outside of cancer treatment facility	
- Tailored on patient characteristics: patient personality, lifestyle, cultural and economic background, age, tumour type, physical and psychosocial status	
Characteristics of patients	
Unaffordable physical activity programme	
- Patient cannot finance physical activity programme	
Too little knowledge about their own health and healthcare process	
No responsibility for their own health	
Characteristics of professionals	
PHPs without enough knowledge and skills about physical activity programmes	
PHPs expecting extra work	
- PHPs expecting more time pressure due to extra work	
Non-committed HCPs	
- HCPs do not expect benefit for the patient	
HCPs disliking new approaches (shared survivor-care system)	
Characteristics of the social setting and context in which the physical activity programme has to be applied	
Negative influence of social network on patient	
Characteristics of the organisation	
Not enough cooperation between healthcare institutes	
Difficulties reaching physical activity programme contact persons	
Insufficient quality insurance	
Not enough networking	
Not enough coordination	
Inadequate triage system	
Physical activity programme offered at wrong time in the treatment schedule	
Physical activity programme scheduled at the wrong place and time	
Not enough information about physical activity programmes available to patient	
Limited capacity for delivering physical activity programmes	
- Limited capacity of financial sources, facilities and materials	
- Limited capacity of HCPs	
Characteristics of the laws and governance	
Unclear insurance	

*HCP* healthcare professional, *PHP* primary healthcare professional, *SHP* secondary healthcare professional

### Domain of physical activity programmes

Both PHPs and SHPs said that they believed that physical activity programmes should be tailored to the needs of the patient. They also indicated that inadequate evidence about the effects of physical activity programmes was an impediment to their use. Additionally, PHPs said that the absence of consensus on these programmes also impeded their use. They pointed out that contact with peers who had experience in dealing with cancer and its treatments was a facilitator that encouraged patients to join a physical activity programme. Moreover, PHPs thought that clearly indicating the goals of the physical activity programmes could also help in the implementation of these programmes. Quotes illustrating the barriers and facilitator in the domain of physical activity programmes are:

“Tailored care. Really for the individual patient.”

“I think that that offers possibilities for the uncomplicated patients. But now I can imagine some people about whom I think, “That wouldn’t work for them.” But you have to consider that for each patient.”

### Domain of patients

The interviewed PHPs en SHPs expressed their concerns that some patients could not afford the physical activity programmes. The physical activity programme itself might be too costly, but the additional travel expenses and the necessary sports attributes might also be unaffordable. They had thought about the lack of patients’ knowledge about their own health and healthcare process. This might make patients hesitate to participate because of fear of doing more harm than good with such physical activity programme. It might also stop them from fully participating in a shared-care model for cancer survivors. Patients often know little about PHPs’ and SHPs’ responsibilities and duties in the care for patients with and after cancer. The PHPs and SHPs both believed that providing the right information for patients can increase their knowledge. However, some disagreed and said that not all patients were capable of understanding their own health and healthcare process. The PHPs were concerned that patients did not have enough time to participate in a physical activity programme during, but also after their cancer treatment. One quote regarding barriers in the domain of patients is:

“Uh, sometimes I do have the impression that it’s difficult for patients to take responsibility. Some people have left it to others all their lives, as it were; others like medical people or us.”

### Domain of HCPs

Both the PHPs and the SHPs thought that GPs did not have enough knowledge and skills pertaining to the physical activity programmes to screen and successfully refer patients to physical activity programmes. They also felt that non-committed PHPs and SHPs hindered proper screening and referral.

GPs are mainly afraid of extra work if they take over the screening and referral for physical activity programmes in a shared-care model. The SHPs were concerned about PHPs and SHPs having a negative approach towards new thoughts, evidence of physical activity programmes, and a change of care model, such as the shift from the model of hospital care to a survivor shared-care model. The quote below illustrates the barriers in the domain of HCPs:

“What I sometimes also don’t know is... when they ask, “OK, can I take up sports again? Can I start exercising?” Then I say, “Yes.” “Yes, but the gynaecologist said I couldn’t...” So, I really don’t know your views of what patients can or cannot do.”

### Domain of social setting

In the social setting, both the PHPs and SHPs were concerned about the negative influence of the patient’s social network. Peers, partners, family, friends, and neighbours do share their experiences and thoughts with patients, which does not always motivate the patients to join a physical activity programme.

Quotes illustrating this barrier in the social setting are:

“The best thing would be to get the social network of the patient involved in that. A partner with a negative attitude towards physical activity does not always motivate the patient to join”

### Domain of organisation

Most of the PHPs’ and SHPs’ concerns were in the domain of organisation and were mainly about delivering the physical activity programmes in a shared-care model. We also found the biggest discrepancy between PHPs and SHPs in this domain.

The PHPs believed that the cooperation between PHPs and SHPs was not optimal. They explained that this was partly due to insufficient communication of SHPs towards PHPs. Especially the GPs felt that they were not involved, or involved too late, in the healthcare of their patients with cancer. Another challenge was contacting the SHPs and receiving the information about the patient’s cancer history. Because there was no consensus about the roles in the survivor care, they could not fully cooperate in a shared-care model. Many PHPs were not sure whether the SHPs delivered proper survivor care, but they had no clear role for taking the responsibility to deliver it themselves.

When talking about cooperation and referral, many PHPs raised their concerns about inadequate networking with each other and with SHPs. They also had concerns about the quality assurance of the physical activity programmes. These circumstances made the GPs hesitate to cooperatively communicate and refer. Deliverers of physical activity programmes in primary care stated that the lack of a good network for GPs, SHPs, and each other impeded the referral of patients to them.

Remarkably, the SHPs only raised their concerns about inadequate cooperation and networks between healthcare institutes. They did not raise any concerns about poor cooperation or communication between themselves and the PHPs. Representative quotes that capture these barriers are:

“The boomerang effect! With the best of intentions, you refer a patient and then you get him back straight away… You can avoid that by having a case manager.”

“Uh hmm. Yes I think that, especially in the multidisciplinary case, it is really important and that there should just be more reciprocal communication.”

“That we know: in this discipline this is done, that discipline looks after that task, etc., that we tell each other what we are doing. So that the island feeling that we have now will disappear.”

“If you don’t continue the same policy, via the GP, then it could just be another wall for the patient.”

### Domain of law and governance

Most of the PHPs and SHPs recognised that it was unclear whether and what parts of the physical activity programmes were insured. There was too much variety of reimbursement, which made reimbursement unclear for both HCPs and patients. The HCPs could not fully inform the patients about the costs. They felt that this obscure reimbursement system eventually stopped patients from joining physical activity programmes. One quote illustrating the unclear insurance-coverage as a barrier is:

“The insurance is very important. Because the insurance does not cover a great many things. And it’s really a horrible job for us to get everything all done.”

## Discussion

In this study with PHPs and SHPs involved in the screening, referral, and delivery of physical activity programmes in a shared-care model for survivors, we identified multiple barriers that hinder implementation. A physical activity programme that was not tailored to the patient impeded implementation, as did the inadequate knowledge and skills about physical activity programmes and the non-commitment of the HCPs. HCPs expressed their concerns about the negative influence of the patient’s social network. Most barriers occurred in the domain of organisation. The HCPs were particularly concerned about the quality of collaboration and networks between hospitals. The insurance coverage was inadequate for physical activity programmes and information about what was covered was elusive. The PHPs raised concerns about lack of communication, unclear roles, and little collaboration with the SHPs, while, surprisingly, the SHPs did not raise such concerns.

An interesting finding was that all HCPs expressed their concerns about the negative influence of the patient’s social network. More than 80% of cancer patients obtain treatment information and support from their social network [28, 29]. This network helps patients in understanding information, provides support and manages overall care that patients would otherwise not receive from their HCPs [28]. Most patients start and continue to do physical activities due to the emotional and practical support of their social network [30, 31]. One should note that physical activity is still rarely a topic in daily clinical practice and patients may seek support from their social network. These findings may therefore be of importance and should lead to physical activity being included as a regular topic that needs attention in the regular care for cancer patients and survivors.

Surprisingly, we found that PHPs raised concerns about lack of communication, unclear roles, and scant collaboration with the SHPs, while the SHPs themselves did not raise such concerns. In other studies, lack of collaboration and miscommunication between PHPs and SHPs was also found, in both directions [32–36]. SHPs assigned this to the large number of PHPs, their varied level of commitment and knowledge, lack of time, and difficulties in contacting them [33]. PHPs thought this was due to lacking communication of patients’ treatment information as well as SHPs who were not available for consultation when needed [34].

Concerns existed about the sustainability of the traditional model of delivering care to cancer survivors in secondary care. Sharing the delivery of this care with PHPs was seen as a solution. No differences were found in the quality of life or patient satisfaction [37], but survivors in the care of PHPs tend to receive more preventive care and appropriate care for co-morbidities. They have greater patient satisfaction than do survivors in the care of SHPs alone [38, 39]. There are also cost reductions due to fewer hospital appointments [40]. Nevertheless, most PHPs are less informed about the patients’ cancer diagnoses and treatment history. They have had few experiences with cancer patients; they may be unaware of potential long-term complications of cancer and its treatment, and may be unprepared to handle them [41–43]. Our findings confirm that few PHPs and SHPs prefer a shared-care model for cancer survivors [17–21]. PHPs hesitate to take responsibility in this model because of their lack of experience with cancer patients and limited training in survivor care [18–21]. PHPs are also reluctant to accept such responsibilities because of the limited time they have to address cancer survivors’ needs [19, 44]. From their perspective, SHPs are worried about the knowledge and skills of the PHPs for delivering proper screening, referral, and physical activity programmes [45]. The PHPs we interviewed explained that these concerns were enlarged by lack of communication, vague roles, poor networks, and little collaboration with the SHPs.

A shared-care model ensures that patients receive survivor care throughout treatment and along the continuum of cure and palliation. It puts PHPs and SHPs on the same team. Several studies report that team-based cancer care can improve patient outcomes and the quality of healthcare [46]. Some characteristics of a healthcare team that works well are: shared goals, clear roles, trust in each other, effective communication, leadership, and measurable processes and outcomes [47, 48]. Our data and the literature show that these factors need improvement, especially the factors for clear roles, trust in each other, oneself, and effective communication.

Our data indicate the need of some on-going initiatives to facilitate implementation of physical activity programmes in a shared-care model. These initiatives include the use of survivor care plans, improving rehabilitation guidelines, improving networks, and the use of health information technology. During our interviews, the PHPs raised their concerns about lacking knowledge and little cooperation and communication with SHPs. Although little is yet known about the effects of survivor-care plans on patients outcomes, it is clear that such plans help PHPs provide survivor care with more confident [19, 21, 22, 49, 50]. They can also improve communication with SHPs, while providing PHPs with the necessary information.

Guidelines for rehabilitating patients with cancer are written quite generically. They do not give explicit recommendations or guidance about roles and responsibility. A guideline giving more specific guidance about what, when, which patient, which HCP, and why can guide both SHPs and PHPs to providing better care.

Health information technology can serve as a tool to facilitate efficiencies in shared-care. It can help to rapidly collect, share, and analyse data in an accessible, actionable, timely, customisable, and portable way between PHPs and SHPs [51]. Health information technology can help create networks for easy communication with qualified HCPs. It can help deliver easy access to patient records and allow HCPs to see each other’s notes. Health information technology can also assist in clarifying roles by sending reminders to the right HCPs. It may help measure the quality of healthcare processes and outcomes. Several characteristics of health information technology can also help improve patient and caregiver engagement by providing patients’ online access to their medical records, clinicians’ notes, care plans, relevant clinical information about their health status, etc. [52]. Additionally, patients’ self-reported health status, side effects of treatment, and sharing of other experiences as they occur [53] can help them become part of the shared-care team.

Implementing physical activity programmes in a shared-care model requires changes at the policy level. Much literature focuses on the shortfall of SHPs in cancer care [11, 12]. However, like the data in our study, other data show that PHPs do not have the capacity to manage the influx of cancer survivors who need physical activity programmes that would occur if the responsibility were transferred from a traditional SHP survivor-care model to a shared-care model [45, 54].

Other reasons for political changes are the insufficient insurance coverage, uncertainty and lack of information on the insurance coverage of physical activity programmes. Our current study suggests that insufficient and uncertain insurance coverage probably affects the implementation of physical activity programmes; this seems to be related as well to a lack of evidence regarding the most cost effective approaches. This requires better awareness, better evidence and advocacy of patients and professionals to better arrange reimbursement of physical activity programmes in cancer care. Providing survivors and their HCPs with clear information about insurance coverage and alternatives for the programmes with financial hurdles is an essential first step.

Our study has several strengths and limitations. The strength of qualitative methodology is that it can deliver a deeper understanding of the thought and experiences of individual HCPs [27, 55]. However, findings derived in a qualitative approach are harder to generalise to the broader population than findings derived in a quantitative approach.

We conducted 4 focus group interviews with 7, 7, 12 and 13 participants. Focus group interviews with more than 10 participants are difficult to control and they limit each person’s opportunity to share insights and observations. In addition, group dynamics change when participants want to, but aren’t able to describe their experiences. However group sizes can have as many as 12–15 participants when there is a good moderator. We used an experienced moderator and experienced no problems concerning control and opportunities to share insights.

Our exploratory and qualitative approach to the focus group interviews of SHPs has the disadvantage of identifying the barriers and facilitators of the entire group of SHPs. This makes it hard to distinguish which differences exist in the views of the various professionals throughout the hospital (e.g. surgeons, radiotherapists, medical oncologists, gynaecologists, urologists, rehabilitation physicians, sports-medicine physicians, physiotherapists, physician assistants, nurses and psychologists). Explorative studies performed among the different professionals separately would be a way of showing the contrast of barriers and facilitators between these different disciplines.

The HCPs in our study represented a diversity of hospitals in different regions in the Netherlands and may not necessarily reflect the situation of cancer survivor care in other countries. The incentive to start physical activity programmes might be different in other countries with different healthcare systems and often even more limited reimbursement policies. Although more research is needed to assess the nature of barriers and facilitators of physical activity programme implementation in other countries, our personal impression is that the findings may well be transferred to other countries.

Although the results of this study seem partly confirmatory, we think it adds relevant information, especially in view of the lagging implementation rates of physical activity programmes and scarce material on approaches to implement these programmes [56–59]. We are in need of more detailed and personalised suggestions for improvement. Results of a qualitative study that gives insight into potential barriers and facilitators [60] to tailor implementation strategies can thus be very helpful.

## Conclusion

Most barriers to implementing physical activity programmes in a shared-care model for cancer survivors are in the domain of organisation: the collaboration, communication, networks and clear roles between PHPs and SHPs are currently inadequate. Survivor care plans, improved rehabilitation guidelines, smoothly functioning networks and more use of health information technology would all facilitate implementation. The knowledge gathered in this study can be used to develop a successful strategy for implementing physical activity programmes and improve the quality of cancer care.

## Electronic supplementary material

ESM 1 (DOCX 31 kb)

## Abbreviations

HCP: Healthcare professional
PHP: Primary healthcare professional
SHP: Secondary healthcare professional
