# Supplementary material for: Healthcare professionals’ perspectives of barriers and facilitators in implementing physical activity programmes delivered to cancer survivors in a shared-care model: a qualitative study
Source: Support Care Cancer. 2019 Dec 2;28(7):3429–40. doi: 10.1007/s00520-019-05108-1 (PMC7256088; doi:10.1007/s00520-019-05108-1)
Supplement: Supplementary file 1 — (DOCX 31 kb) [file 520_2019_5108_MOESM1_ESM.docx]

**Consolidated criteria for reporting qualitative studies (COREQ): 32-item checklist**

Developed from:

Tong A, Sainsbury P, Craig J. Consolidated criteria for reporting qualitative research (COREQ): a 32-item checklist for interviews and focus groups. International Journal for Quality in Health Care 2007. Volume 19, Number 6: pp. 349 –357

| **No** | **Item** | **Guide questions/description** |  |
| --- | --- | --- | --- |
| **Domain 1: Research team and reflexivity** | | |  |
| Personal Characteristics | |  |  |
| 1. | Interviewer/facilitator | Which author/s conducted the interview or focus group? | *Individual interviews: Charlotte IJsbrandy*  *Focus-group interviews: Charlotte IJsbrandy and Rosella Hermens* |
| 2. | Credentials | What were the researcher's credentials? *E.g. PhD, MD* | *Charlotte IJsbrandy, MD*  *Rosella Hermens, PhD* |
| 3. | Occupation | What was their occupation at the time of the study? | *Charlotte IJsbrandy, Researcher*  *Rosella Hermens, Associate Professor* |
| 4. | Gender | Was the researcher male or female? | *Both female* |
| 5. | Experience and training | What experience or training did the researcher have? | *Charlotte IJsbrandy took a course in qualitative research methods*  *Rosella Hermens has years of experience with focus-group interviews* |
| Relationship with participants | |  |  |
| 6. | Relationship established | Was a relationship established prior to study commencement? | *No for both* |
| 7. | Participant knowledge of the interviewer | What did the participants know about the researcher? e*.g. personal goals, reasons for doing the research* | *All participants received written information stating the objectives and the process of the individual or focus-group interview. Before the interviews began, the participants were orally informed about the objectives, process of the individual or focus-group interview, process after the individual or focus-group interview, and both researchers’ goals and reasons for doing the research.* |
| 8. | Interviewer characteristics | What characteristics were reported about the interviewer/facilitator? e.g. *Bias, assumptions, reasons and interests in the research topic* | *The background of the interviewers.* |
| **Domain 2: study design** | | |  |
| Theoretical framework | |  |  |
| 9. | Methodological orientation and Theory | What methodological orientation was stated to underpin the study? *e.g. grounded theory, discourse analysis, ethnography, phenomenology, content analysis* | *Content analysis* |
| Participant selection | |  |  |
| 10. | Sampling | How were participants selected? *e.g. purposive, convenience, consecutive, snowball* | *Individual interviews were conducted with professionals involved in treating cancer patients in primary care and practicing in the regions of five participating hospitals (e.g. general practitioners and physiotherapists). They were asked to participate by letter and could reply to accept .*  *Four hospitals participated in the focus-group study. Ten to 12 professionals involved in treating cancer patients in secondary care were invited (e.g. surgeons, radiotherapists, medical oncologists, gynaecologists, urologists, rehabilitation physicians, sports-medicine physicians, physiotherapists, physician assistants, and psychologists). Secondary healthcare professionals were asked to participate by letter, to which they could reply.* |
| 11. | Method of approach | How were participants approached? e*.g. face-to-face, telephone, mail, email* | *Participants were asked by letter.* |
| 12. | Sample size | How many participants were in the study? | *31 participants in the individual interviews and 39 in the focus-group interviews* |
| 13. | Non-participation | How many people refused to participate or dropped out? Reasons? | *None of the patients who were willing to participate dropped out.* |
| Setting |  |  |  |
| 14. | Setting of data collection | Where was the data collected? e*.g. home, clinic, workplace* | *Participants’ clinics* |
| 15. | Presence of non-participants | Was anyone else present besides the participants and researchers? | *No* |
| 16. | Description of sample | What are the important characteristics of the sample? *e.g. demographic data, date* | *Individual interviews were conducted with primary healthcare professionals involved in treating cancer patients in primary care (e.g. general practitioners and physiotherpists).*  *For the focus-group interviews, secondary healthcare professionals involved in the treatment of cancer patients in secondary care were invited (e.g. surgeons, radiotherapists, medical oncologists, gynaecologists, urologists, rehabilitation physicians, sports-medicine physicians, physiotherapists, physician assistants, and psychologists).* |
| Data collection | |  |  |
| 17. | Interview guide | Were questions, prompts, guides provided by the authors? Was it pilot tested? | *Interview guides were developed from Grol and Flottorp’s theoretical models to identify influencing factors.* |
| 18. | Repeat interviews | Were repeat interviews carried out? If yes, how many? | *31 individual interviews and four focus-group interviews.* |
| 19. | Audio/visual recording | Did the research use audio or visual recording to collect the data? | *The interviews were audio recorded.* |
| 20. | Field notes | Were field notes made during and/or after the interview or focus group? | *If applicable, notes were made.* |
| 21. | Duration | What was the duration of the interviews or focus group? | *The individual interviews took about 30 minutes each. The focus group interviews took about 90 minutes each.* |
| 22. | Data saturation | Was data saturation discussed? | *Yes* |
| 23. | Transcripts returned | Were transcripts returned to participants for comment and/or correction? | *No* |
| **Domain 3: analysis and findings** | | |  |
| Data analysis | |  |  |
| 24. | Number of data coders | How many data coders coded the data? | *Two: Charlotte IJsbrandy, MD, Researcher*  *Laura Boerboom, MSc, Researcher* |
| 25. | Description of the coding tree | Did authors provide a description of the coding tree? | *The factors identified were classified within the earlier mentioned frameworks of Grol and Flottorp. We used the following domains: 1)* *characteristicsof the physical activity programmes, 2) characteristics of the professionals, 3) characteristics of the patients, 4) characteristics of the social setting, 5) characteristics of the organization and 6) characteristics of law and governance.* |
| 26. | Derivation of themes | Were themes identified in advance or derived from the data? | *The factors identified were classified within Grol and Flottorp’s frameworks. Factors that had been identified, but had not been present in our model, were added. The two investigators discussed their interpretation until consensus was reached.* |
| 27. | Software | What software, if applicable, was used to manage the data? | *Atlas.ti version 7.6.16.* |
| 28. | Participant checking | Did participants provide feedback on the findings? | *No* |
| Reporting |  |  |  |
| 29. | Quotations presented | Were participant quotations presented to illustrate the themes / findings? Was each quotation identified? e*.g. participant number* | *Yes* |
| 30. | Data and findings consistent | Was there consistency between the data presented and the findings? | *Yes* |
| 31. | Clarity of major themes | Were major themes clearly presented in the findings? | *Yes* |
| 32. | Clarity of minor themes | Is there a description of diverse cases or discussion of minor themes? | *Yes* |
